# Effects of post-exercise compression garments on subsequent endurance performance and delayed-onset muscle soreness in endurance athletes: a systematic review and meta-analysis

**DOI:** 10.3389/fphys.2026.1908148

**Published:** 2026-07-31

**Authors:** Magdalena Hagner-Derengowska, John Xerri de Caro, Zofia Dziecioł-Anikiej, Agnieszka Sozańska, Duško Bjelica, Carla Gonçalves, Robert Trybulski

**Affiliations:** 1 Sport Research Center, Faculty of Earth Sciences and Spatial Management, Nicolaus Copernicus University, Torun, Poland; 2 Department of Physiotherapy, Faculty of Health Sciences, University of Malta, Msida, Malta; 3 Physical Activity Research Laboratory, Clinical Research Center, Department of Personalized Physiotherapy of the Musculoskeletal System, Medical University of Białystok, Białystok, Poland; 4 Collegium Medicum, Faculty of Health Sciences and Psychology, Institute of Physiotherapy, University of Rzeszów, Rzeszów, Poland; 5 University Center for Research and Development in Health Sciences, Laboratory for Research on Health, Functioning and Disability, University of Rzeszów, Rzeszów, Poland; 6 Faculty of Sport and Physical Education, University of Montenegro, Nikšić, Montenegro; 7 Montenegrian Sports Academy, Podgorica, Montenegro; 8 Escola Superior de Desporto e Lazer, Instituto Politécnico de Viana do Castelo, Viana do Castelo, Portugal; 9 The Sport Physical Activity and Health Research & Innovation Center, Viana do Castelo, Portugal; 10 Faculty of Medicine, Katowice Business University, Katowice, Poland; 11 Provita Żory Medical Center, Żory, Poland

**Keywords:** athletes, athletic performance, compression clothing, compression garments, delayed-onset muscle soreness, exercise recovery, physical endurance

## Abstract

**Objective:**

To evaluate whether post-exercise compression garments improve subsequent endurance performance and delayed-onset or perceived muscle soreness in adult endurance athletes.

**Methods:**

This systematic review and meta-analysis searched PubMed, Scopus, and Web of Science through 19 May 2026 for controlled intervention studies comparing post-exercise compression garments with no compression, usual clothing, sham/placebo, passive recovery, or lower-pressure controls. RoB 2 and GRADE assessed risk of bias and certainty. Soreness was synthesized as Hedges g using random-effects models with Hartung-Knapp-Sidik-Jonkman inference; performance was synthesized narratively. The protocol was registered in Open Science Framework (osf.io/46ck9).

**Results:**

Of 503 records screened, nine studies were included (158 participants; six crossover, three parallel-group). All studies included in the final evidence base were randomized parallel-group or crossover trials. Ten of 11 assessments were high risk. At approximately 24 hours, soreness favored compression but was uncertain (four studies; Hedges g = 0.62, 95% CI -0.57 to 1.82; I^2^ = 74.0%). Immediate-to-2-hour acute post-exercise perceived soreness/discomfort was also uncertain (three studies; g = 0.38, 95% CI -0.67 to 1.42; I^2^ = 51.0%). Performance findings were not pooled because of heterogeneity or non-isolatable recovery effects. Certainty was very low.

**Conclusions:**

Soreness estimates were directionally favorable to compression, but the evidence was very uncertain, statistically imprecise, and vulnerable to expectation effects because blinding was limited or poorly credible. Effects on subsequent endurance performance remain unresolved. Registration: OSF osf.io/46ck9 on 14/05/2026.

**Systematic Review Registration:**

https://osf.io/b3c78/overview, identifier osf.io/46ck9.

## 1 Introduction

Endurance athletes often require recovery between repeated training or competition exposures, and recovery research in endurance sport evaluates restoration of functional performance as well as perceptual responses after exercise (Li et al., 2024). Compression garments are elastic garments worn during or after exercise to apply external pressure to body regions, and their sport applications have included performance support, fatigue attenuation, and post-exercise recovery (Weakley et al., 2022). These applications should be distinguished by timing and intended effect since garments worn during exercise are usually evaluated for acute performance, biomechanical, thermoregulatory, or perceptual effects, whereas garments worn after exercise are evaluated as recovery interventions intended to influence subsequent performance or post-exercise symptoms (Weakley et al., 2022). The compression-garment literature is methodologically heterogeneous because studies differ in exercise mode, garment coverage, applied pressure, timing of use, participant characteristics, and outcome domain (Weakley et al., 2022).

Proposed mechanisms include changes in muscle oscillation, sensorimotor feedback, local skin temperature, blood-flow responses, and perceptions of soreness or pain, but consistent cardiorespiratory and metabolic effects have not been established (Weakley et al., 2022). Post-exercise compression has been investigated in relation to both objective recovery of performance and subjective symptoms such as delayed-onset muscle soreness (Hill et al., 2014b; Brown et al., 2017). In the context of our study, soreness is used as an umbrella term for self-reported muscle soreness after exercise. Delayed-onset muscle soreness (DOMS) refers more specifically to soreness that emerges and intensifies after a delay, commonly peaking after approximately 24 to 48 hours, whereas early post-exercise ratings may reflect acute perceived soreness or discomfort rather than classic DOMS (Vickers, 2001). For endurance athletes, subsequent endurance performance and muscle soreness are particularly relevant because they respectively reflect functional recovery and athlete-perceived recovery burden after training or competition (Engel et al., 2016; Brown et al., 2017).

A 2017 meta-analysis assessed compression garments for recovery of strength, power, and endurance performance after resistance, running, or non-load-bearing endurance exercise and extracted data from 23 studies (Brown et al., 2017). A runner-focused review reported possible benefits for variables related to endurance performance and for post-exercise soreness, but it addressed compression clothing in runners rather than a strictly post-exercise recovery intervention (Engel et al., 2016). A 2025 updated meta-analysis on compression garments during running did not provide evidence supporting improvements in race time or time to exhaustion (Wang et al., 2025). A 2025 systematic review with meta-analysis of compression socks during running reported very low- to moderate-certainty evidence that compression socks did not benefit physiological, running-performance, or perceptual outcomes compared with regular socks (Telles et al., 2025). These prior syntheses are informative, but they include different intervention timings, exercise modalities, populations, and outcome domains. In particular, combining garments worn during exercise, after exercise, and during subsequent testing can obscure whether any observed effect reflects recovery, an acute in-exercise mechanical or perceptual effect, or a mixed-timing effect (Engel et al., 2016; Brown et al., 2017; Weakley et al., 2022; Wang et al., 2025).

Existing syntheses are informative but commonly combine different exercise modalities, garment timings, participant populations, and outcome domains (Hill et al., 2014b; Brown et al., 2017; Weakley et al., 2022). The endurance-athlete recovery literature has also identified inconsistency and limited evidence for making general recommendations about recovery strategies, including compression garments (Li et al., 2024). These limitations justify a focused review that separates post-exercise compression use from garments worn during exercise and that prespecifies subsequent endurance performance and delayed-onset muscle soreness as the main outcomes (Brown et al., 2017; Weakley et al., 2022).

This systematic review and meta-analysis aims to evaluate the effects of post-exercise compression garments, compared with no compression, usual clothing, sham compression, or passive recovery, on subsequent endurance performance and delayed-onset muscle soreness in endurance athletes. The review aims also to examine whether effects differ according to recovery time window, endurance sport modality, garment coverage, applied pressure, comparator type, and exercise-damage characteristics. The distinguishing contribution of this review is not that compression garments have been unreviewed previously, but that it applies a recovery only, endurance-athlete-focused context that separates post-exercise compression from in-exercise or mixed-timing compression use. This narrower background prioritizes outcomes that are directly relevant to recovery practice, i.e., subsequent endurance performance and delayed-onset or perceived muscle soreness.

## 2 Methods

The review was written in accordance with PRISMA 2020 (Page et al., 2021). The search methods were reported using PRISMA-S (Rethlefsen et al., 2021). The review question was: In endurance athletes, do compression garments worn after exercise improve subsequent endurance performance and reduce delayed-onset muscle soreness compared with no compression, usual clothing, sham compression, or passive recovery? The study protocol had been prospectively registered on the Open Science Framework platform (ID: osf.io/46ck9; Date: 14/05/2026). PROSPERO was not used as the registration platform. Published systematic reviews were identified through the database search, reference-list checking, and forward citation searching, but PROSPERO and other review registries were not part of the original search strategy for overlapping registered reviews. No deviations from the core OSF protocol question, eligibility criteria, co-primary outcomes, or main synthesis framework were made.

### 2.1 Eligibility criteria

#### 2.1.1 Population

Eligible participants were healthy adult endurance athletes or endurance-trained adults aged 18 years or older. Endurance athletes included, but were not limited to, runners, cyclists, triathletes, cross-country skiers, rowers, swimmers, and other athletes whose primary sport was predominantly endurance-based. Studies including mixed athlete samples were eligible only if data for endurance athletes were available separately. Studies conducted exclusively in clinical populations, injured participants, children or adolescents, occupational samples, or untrained sedentary adults were excluded unless separate eligible endurance-athlete data could be extracted.

#### 2.1.2 Intervention

Eligible interventions were static wearable compression garments used during the post-exercise recovery period. Eligible garments included compression socks, stockings, calf sleeves, tights, pants, shorts, lower-body garments, upper-body garments, or full-body garments, provided that they were applied after the exercise stimulus and intended to apply external pressure during recovery. Studies in which compression was worn only during exercise were excluded. Studies in which compression garments were worn during both exercise and post-exercise recovery were eligible for the overall review only when the post-exercise garment-use phase was clearly described and at least one eligible recovery outcome was reported. However, such mixed-timing studies were eligible for recovery-only quantitative synthesis only when the design allowed the post-exercise recovery effect to be isolated from any in-exercise garment exposure. If the garment was worn during the initial exercise bout, during the subsequent performance test, or during both recovery and subsequent testing, and the independent post-exercise recovery effect could not be separated, the study was retained for narrative synthesis only and was flagged as a mixed-timing design. Studies evaluating intermittent pneumatic compression devices, compression boots, massage devices, kinesiology tape, bandages, medical compression for disease management, or multi-component recovery protocols were excluded unless the independent effect of the compression garment could be separated from the co-intervention. Garment pressure, pressure-gradient characteristics, body coverage, material, manufacturer, size-fitting procedure, wear duration, wear timing, and participant adherence were extracted whenever reported. If pressure was not reported, the study remained eligible but was flagged as having an incomplete intervention description.

#### 2.1.3 Comparators

Eligible comparators included no compression, usual clothing, standard recovery, passive recovery, placebo or sham garments, or lower-pressure garments that the original investigators intended as control conditions. Active recovery modalities such as cold-water immersion, massage, stretching, electrical stimulation, or nutritional supplementation were not accepted as sole comparators unless both study arms received the same co-intervention and the only systematic difference between groups was the compression garment. In crossover studies, the comparator period was treated as the within-participant control condition when the washout period was considered adequate for the outcome being analyzed.

#### 2.1.4 Outcomes

Studies were required to report at least one of the two co-primary outcomes to be eligible for inclusion. Contribution to quantitative synthesis additionally required sufficient numerical data and at least three sufficiently comparable studies within the same outcome domain and recovery-time window. The first co-primary outcome was subsequent endurance performance after the recovery period. Eligible performance outcomes included time-trial time, distance completed in a fixed time, time to exhaustion, mean power output, mean speed or pace, repeated endurance-test performance, or an equivalent endurance-specific performance measure assessed after the recovery intervention. For contribution to recovery-only quantitative synthesis, the outcome also had to reflect a contrast in which the effect of post-exercise compression could be isolated. Performance outcomes assessed while participants continued to wear the compression garment were not treated as recovery-only outcomes unless the study design separated the post-exercise recovery effect from the acute effect of wearing the garment during performance testing. Outcomes measured during the initial exercise bout were not treated as recovery outcomes unless the design explicitly tested performance in a subsequent exercise bout after a recovery interval. The second co-primary outcome was delayed-onset muscle soreness or perceived muscle soreness after exercise, measured using a visual analogue scale, numerical rating scale, Likert-type scale, or another self-report soreness scale.

The main recovery time window for both co-primary outcomes was approximately 24 hours post-exercise, defined pragmatically as 20 to 30 hours after the exercise stimulus when such timing was available. Additional prespecified windows were immediate to 2 hours, more than 2 to 8 hours, more than 8 to less than 20 hours, more than 30 to 48 hours, more than 48 to 72 hours, and more than 72 hours after exercise. When multiple observations were available within the same time window, the measure closest to the target time point was used for the primary synthesis. Laboratory, field, and competition-simulation settings were eligible. Studies conducted during actual competition were eligible if the post-exercise compression period and subsequent outcome assessment were clearly defined.

#### 2.1.5 Study designs

Eligible randomized study designs included randomized parallel-group trials, randomized crossover trials, and cluster-randomized trials. Non-randomized controlled intervention studies were eligible only if they contained a clearly defined comparator group or comparator condition and the post-exercise compression effect could be separated from co-interventions or mixed garment timing. Such studies were planned to be analyzed separately or included only in sensitivity analyses depending on risk of bias, clinical comparability, and ability to isolate the recovery effect; they were not planned to be combined with randomized trials in the main certainty rating. Uncontrolled before-after studies, quasi-experimental studies without a clearly defined comparator, case reports, case series, narrative reviews, systematic reviews, conference abstracts without sufficient data, editorials, letters without original data, and animal or mechanistic bench studies were excluded. In the final evidence base, all included studies were randomized parallel-group or randomized crossover trials.

### 2.2 Information sources

Electronic searches were conducted on 19 May 2026 in PubMed, Scopus, and Web of Science. No date restrictions were applied. No language restrictions were applied at the search stage. Non-English records were translated where feasible, and any full texts that could not be translated were listed with the reason for exclusion. Reference lists of eligible studies and relevant reviews were checked manually. Forward citation searching of eligible studies and key reviews was performed in PubMed, Scopus, and Web of Science.

### 2.3 Search strategy

The search strategy was developed around three concepts: compression garments; endurance exercise or endurance athletes; and post-exercise recovery, subsequent endurance performance, or muscle soreness. **Table 1** details the search syntax in each database.

**Table 1:** Search syntax in each database.

Database	Search syntax
PubMed	[“Elastic Stockings”(Title/Abstract) OR “compression garment”(Title/Abstract) OR “compression garments”(Title/Abstract) OR “compression clothing”(Title/Abstract) OR “compression apparel”(Title/Abstract) OR “compression sock”(Title/Abstract) OR “compression socks”(Title/Abstract) OR “compression stocking”(Title/Abstract) OR “compression sleeve”(Title/Abstract) OR “compression pants”(Title/Abstract) OR “compression shorts”(Title/Abstract) OR “graduated compression”(Title/Abstract) OR “sports compression”(Title/Abstract) OR “lower-body compression”(Title/Abstract) OR “whole-body compression”(Title/Abstract)] AND [“endurance”(Title/Abstract) OR “aerobic exercise”(Title/Abstract) OR “physical endurance”(Title/Abstract) OR “running”(Title/Abstract) OR “bicycling”(Title/Abstract) OR “athlete”(Title/Abstract) OR “athletes”(Title/Abstract) OR “runners”(Title/Abstract) OR “cycling”(Title/Abstract) OR “triathlon”(Title/Abstract) OR “marathon”(Title/Abstract) OR “distance run”(Title/Abstract) OR “exercise”(Title/Abstract) OR “sports”(Title/Abstract)] AND [“recovery”(Title/Abstract) OR “recover”(Title/Abstract) OR “post-exercise”(Title/Abstract) OR “postexercise”(Title/Abstract) OR “exercise recovery”(Title/Abstract) OR “performance recovery”(Title/Abstract) OR “subsequent performance”(Title/Abstract) OR “time-trial”(Title/Abstract) OR “time-to-exhaustion”(Title/Abstract) OR “time-to-exhaustion”(Title/Abstract) OR “delayed onset muscle soreness”(Title/Abstract) OR “DOMS”(Title/Abstract) OR “muscle soreness”(Title/Abstract) OR “leg soreness”(Title/Abstract) OR “exercise-induced muscle damage”(Title/Abstract) OR “EIMD”(Title/Abstract)]
Scopus	(TITLE-ABS-KEY (“Elastic Stockings” OR “compression garment” OR “compression garments” OR “compression clothing” OR “compression apparel” OR “compression sock” OR “compression socks” OR “compression stocking” OR “compression tight” OR “compression sleeve” OR “compression pants” OR “compression shorts” OR “graduated compression” OR “sports compression” OR “lower-body compression” OR “whole-body compression”) AND TITLE-ABS-KEY (“endurance” OR “aerobic exercise” OR “physical endurance” OR running OR bicycling OR athlete OR athletes OR runners OR cycling OR triathlon OR marathon OR “distance run” OR exercise OR sports) AND TITLE-ABS-KEY (recovery OR recover OR “post-exercise” OR postexercise OR “exercise recovery” OR “performance recovery” OR “subsequent performance” OR “time-trial” OR “time to exhaustion” OR “time-to-exhaustion” OR “delayed onset muscle soreness” OR DOMS OR “muscle soreness” OR “leg soreness” OR “exercise-induced muscle damage” OR EIMD))
Web of Science	“Elastic Stockings” OR “compression garment” OR “compression garments” OR “compression clothing” OR “compression apparel” OR “compression sock” OR “compression socks” OR “compression stocking” OR “compression tight” OR “compression sleeve” OR “compression pants” OR “compression shorts” OR “graduated compression” OR “sports compression” OR “lower-body compression” OR “whole-body compression” (Topic) and “endurance” OR “aerobic exercise” OR “physical endurance” OR running OR bicycling OR athlete OR athletes OR runners OR cycling OR triathlon OR marathon OR “distance run” OR exercise OR sports (Topic) and recovery OR recover OR “post-exercise” OR postexercise OR “exercise recovery” OR “performance recovery” OR “subsequent performance” OR “time-trial” OR “time to exhaustion” OR “time-to-exhaustion” OR “delayed onset muscle soreness” OR DOMS OR “muscle soreness” OR “leg soreness” OR “exercise-induced muscle damage” OR EIMD (Topic)

### 2.4 Study records and data management

All records were exported from each database with full citation information, abstracts, keywords, DOI, and database identifiers whenever available. Records were imported into EndNote online management. Deduplication was performed using automated matching followed by manual verification of potential duplicates. Multiple reports from the same participant dataset were linked under a single study identifier to avoid double counting. When companion reports provided different outcomes or time points from the same dataset, all usable information was extracted, but each participant sample contributed only once to any given meta-analysis. Multilevel and robust-variance models were not used because the conducted meta-analyses retained one effect per study within each outcome and time window.

### 2.5 Selection process

Two authors independently screened titles and abstracts against the eligibility criteria after pilot calibration on a sample of records. Full texts of potentially eligible reports were then assessed independently by two authors. Disagreements at either stage were resolved by discussion or by consultation with a third author. Reasons for exclusion at the full-text stage were recorded and reported. The final selection process was summarized using a PRISMA 2020 flow diagram.

### 2.6 Data collection process

Two authors independently extracted data using a piloted extraction form. Extracted data were compared, and discrepancies were resolved by consensus or by consultation with a third author. When required numerical data were unavailable in tables or text, values were derived from reported statistics or extracted from figures when possible. Figure-derived values were used only when the graph allowed defensible extraction, and the extraction basis was recorded. Data conversions, imputations, and assumptions, including assumed within-participant correlations for crossover studies, were documented.

All values used in quantitative synthesis were classified according to whether they were directly reported, converted from reported uncertainty intervals, derived from reported statistics, extracted from figures, or based on an analytic assumption (**Supplementary Material**). Directly reported arm-level or condition-level means and standard deviations were used without conversion when available. When means were reported with 95% confidence limits rather than standard deviations, standard deviations were derived as SD = confidence-limit half-width × √n/t_{0.975,df}. When only graphical data were available, values were extracted by figure digitization using the WebPlotDigitizer (v3.4, USA). For crossover studies in which within-participant correlations were not reported, the primary sampling variance assumed r = 0.50, with sensitivity analyses using r = 0.25 and r = 0.75.

### 2.7 Data items

The following data items were extracted: study identification details; country and context; study design; participant sex, age, body mass, training status, endurance sport, competition level, and inclusion criteria; sample size by condition; exercise stimulus characteristics, including mode, intensity, duration, gradient or mechanical-damage component when applicable, and environmental conditions; compression-garment type, body coverage, pressure, pressure-gradient profile, material, manufacturer, sizing method, timing of application, duration of wear, adherence, and participant blinding or expectancy procedures; comparator details; co-interventions; washout duration for crossover trials; outcome definitions; measurement instruments; time points; means, standard deviations, change scores, standard errors, confidence intervals, exact p values, paired statistics, and sample sizes; adverse events; and information needed for risk-of-bias assessment. Where available, we also extracted primary-study funding source, garment provenance, and sponsor or manufacturer role, including whether compression garments were purchased by investigators, supplied by manufacturers, provided by external research or textile partners, or not clearly reported. If an outcome was reported with multiple eligible measures, the measures most directly representing the prespecified co-primary outcome were prioritized according to the outcome hierarchy below. Intervention descriptions were extracted using TIDieR-informed items to improve reproducibility of the compression-garment characteristics and co-interventions (Hoffmann et al., 2014).

### 2.8 Outcome hierarchy and prioritization

For subsequent endurance performance, the preferred hierarchy was: standardized time-trial performance in the athlete’s endurance modality; time to exhaustion in a standardized endurance test; distance completed in a fixed-time test; mean power output, speed, or pace during a standardized endurance task; and other endurance-specific performance outcomes judged clinically comparable. For time-based performance outcomes in which lower values indicated better performance, effect directions were reversed so that positive pooled effects consistently favored compression. For DOMS or muscle soreness, the hierarchy prioritized validated or clearly described self-report soreness scales during movement or sport-relevant activity; resting soreness was used when activity-evoked soreness was unavailable. When multiple muscle groups were reported, lower-limb soreness was prioritized for running, cycling, triathlon, and similar lower-limb endurance sports. For the quantitative synthesis of soreness, arm-level follow-up means and standard deviations were used when these were the most consistently available data across studies. Percentage-change values were used for Martínez‐Navarro et al (Martínez‐Navarro et al., 2021). because the results section reported the eligible 24-hour posterior-leg soreness outcome in that form. For crossover trials, paired differences were preferred when reported; when paired change data were unavailable, arm-level values were synthesized using an assumed within-participant correlation with sensitivity analyses.

When a study included multiple compression arms or multiple eligible contrasts, the selected contrast followed a hierarchy. We selected the arm that isolated post-exercise recovery compression from in-exercise compression. Then, when more than one recovery-only compression arm was available, we selected the contrast closest to the review question in timing, outcome window, and comparator type. When multiple eligible recovery-only compression doses remained otherwise comparable, the arm identified by the original authors as the primary compression condition was selected. If no primary arm was specified, the most relevant recovery-dose arm was selected and the alternative arms were summarized narratively. Garment arms worn only during the initial exercise bout, or worn during both recovery and subsequent testing when the recovery-only effect could not be isolated, were not included in recovery-only meta-analysis. No shared-control splitting was required because the conducted meta-analyses retained only one effect per study within each outcome and time window.

### 2.9 Risk of bias assessment

Risk of bias was assessed independently by two authors at the result level for each co-primary outcome. RoB 2 was used for randomized parallel-group and randomized crossover trials, applying the version appropriate to the study design. For crossover trials, special attention was given to randomization of treatment order, deviations from intended interventions, carryover, washout adequacy, missing outcome data, outcome measurement, and selective reporting. If a non-randomized controlled intervention study had met the eligibility criteria, it would have been assessed separately using ROBINS-I and would not have been combined with randomized evidence in the main risk-of-bias summary or main GRADE certainty rating. However, no non-randomized controlled study was included in the final evidence base. Risk-of-bias disagreements were resolved by consensus. Risk-of-bias judgments were incorporated into sensitivity analyses and GRADE certainty assessments rather than being used as automatic exclusion criteria.

### 2.10 Effect measures and data transformations

Continuous outcomes were summarized as standardized mean differences corrected for small-sample bias (Hedges g) when synthesized because soreness scales and reporting formats differed across studies. Effect directions were coded so that positive values indicated benefit from compression, meaning lower delayed-onset or perceived muscle soreness and better endurance performance. For soreness outcomes, where lower values indicated better recovery, the uncorrected standardized mean difference was calculated as d = (M control – M compression)/SD pooled. For time-based performance outcomes in which lower values indicated better performance, signs were reversed so that positive effects consistently favored compression. For parallel-group trials, SD pooled was calculated as: SD pooled = √{[(n compression − 1)SD^2^ compression + (n control − 1)SD^2^ control]/(n compression + n control − 2)}. The small-sample correction was J = 1 − 3/(4df − 1), where df = n compression + n control − 2, and Hedges g = J × d. The sampling variance was calculated as J^2^ × [(n compression + n control)/(n compression n control) + d^2^/(2df)]. For crossover trials, paired effect estimates were preferred when the mean paired contrast and the standard deviation of paired differences were available. When only condition-level follow-up means and standard deviations were available, the point estimate was calculated using the same standardized mean difference structure, with SD pooled = √[(SD^2^ compression + SD^2^ control)/2]. The small-sample correction used df = n − 1. Because the within-participant correlation was usually not reported, the primary crossover sampling variance used an assumed r = 0.50: Var(g) = J^2^ × [2(1 − r)/n + d^2^/(2df)]. Sensitivity analyses used r = 0.25 and r = 0.75. These assumed correlations changed the standard errors and study weights but did not change the condition means, pooled SDs, or Hedges g point estimates. When post-recovery follow-up values were used instead of change scores for consistency across studies, baseline or pre-recovery imbalance was documented in the results table and in the **Supplementary Material**. If standard deviations were unavailable, they were derived from standard errors, confidence intervals, t statistics, p values, reported confidence limits, or figure digitization when possible. No main meta-analysis of endurance performance was conducted because fewer than three studies were sufficiently comparable within a prespecified recovery window and because some performance studies did not isolate the post-exercise recovery effect.

### 2.11 Synthesis methods

A meta-analysis was conducted only when at least three studies were sufficiently comparable in population, intervention, comparator, outcome, and recovery-time window. The primary quantitative synthesis focused on approximately 24 hours post-exercise because this time point was sport-relevant and commonly targeted in recovery studies. Additional time-windowed syntheses were conducted only when at least three sufficiently comparable studies were available. Random-effects models were used as the default because clinical and methodological heterogeneity was expected across endurance sports, compression protocols, comparators, and recovery designs. Between-study variance was estimated using restricted maximum likelihood when possible, and Hartung-Knapp-Sidik-Jonkman (IntHout et al., 2014) inference was used for confidence intervals because small-study meta-analyses were expected in this field.

For each outcome and time-windowed meta-analysis, one effect per study was selected according to the prespecified outcome hierarchy, time-point hierarchy, and multi-arm decision rule. Multilevel meta-analysis and robust variance estimation were not used because the conducted meta-analyses did not retain multiple dependent effects from the same study within the same outcome and time window. Studies or study arms with mixed garment timing were not included in a recovery-only meta-analysis unless the post-exercise compression effect could be isolated from compression worn during the initial exercise bout or during the subsequent performance test. When this isolation was not possible, the result was summarized narratively. Separate reports of the same participant sample were not treated as independent studies within the same meta-analysis.

If quantitative synthesis was inappropriate, findings were summarized narratively using structured synthesis principles, with emphasis on direction of effect, precision, consistency, risk of bias, comparator credibility, isolation of the recovery effect, and clinical applicability. Subgroup analyses, meta-regression, and prediction intervals were not conducted because no meta-analysis included enough studies for reliable estimation.

### 2.12 Assessment and exploration of heterogeneity

Statistical heterogeneity was quantified using tau-squared and I-squared, and Cochran’s Q was reported with appropriate caution because of limited power when the number of studies was small. Prediction intervals were not reported because no meta-analysis included at least 10 studies. Clinical and methodological heterogeneity was assessed before pooling by comparing population training status, endurance modality, exercise stimulus, garment pressure, coverage, wear duration, comparator type, outcome definition, and timing. Large heterogeneity was not interpreted solely as a statistical problem, but also as potential evidence that intervention effects varied by garment protocol, sport context, or recovery demand.

### 2.13 Subgroup and meta-regression analyses

Subgroup analyses and meta-regression were not conducted because no meta-analysis included at least 10 studies. Prespecified candidate moderators, including endurance sport modality, post-exercise wear duration, garment pressure, recovery duration before outcome testing, and exercise-stimulus duration, were therefore summarized descriptively only.

### 2.14 Certainty of evidence

The certainty of evidence was assessed using GRADE (Balshem et al., 2011; Guyatt et al., 2011) for each co-primary outcome at the main time point and, if data permitted, at 48 and 72 hours. Randomized trials started as high-certainty evidence and could be rated down for risk of bias, inconsistency, indirectness, imprecision, and publication bias. If eligible non-randomized controlled evidence had been identified, it would have been rated separately according to GRADE principles and would not have been combined with randomized evidence in the main certainty rating. The reported certainty ratings are based on randomized-trial evidence.

## 3 Results

### 3.1 Study selection

**Figure 1:**
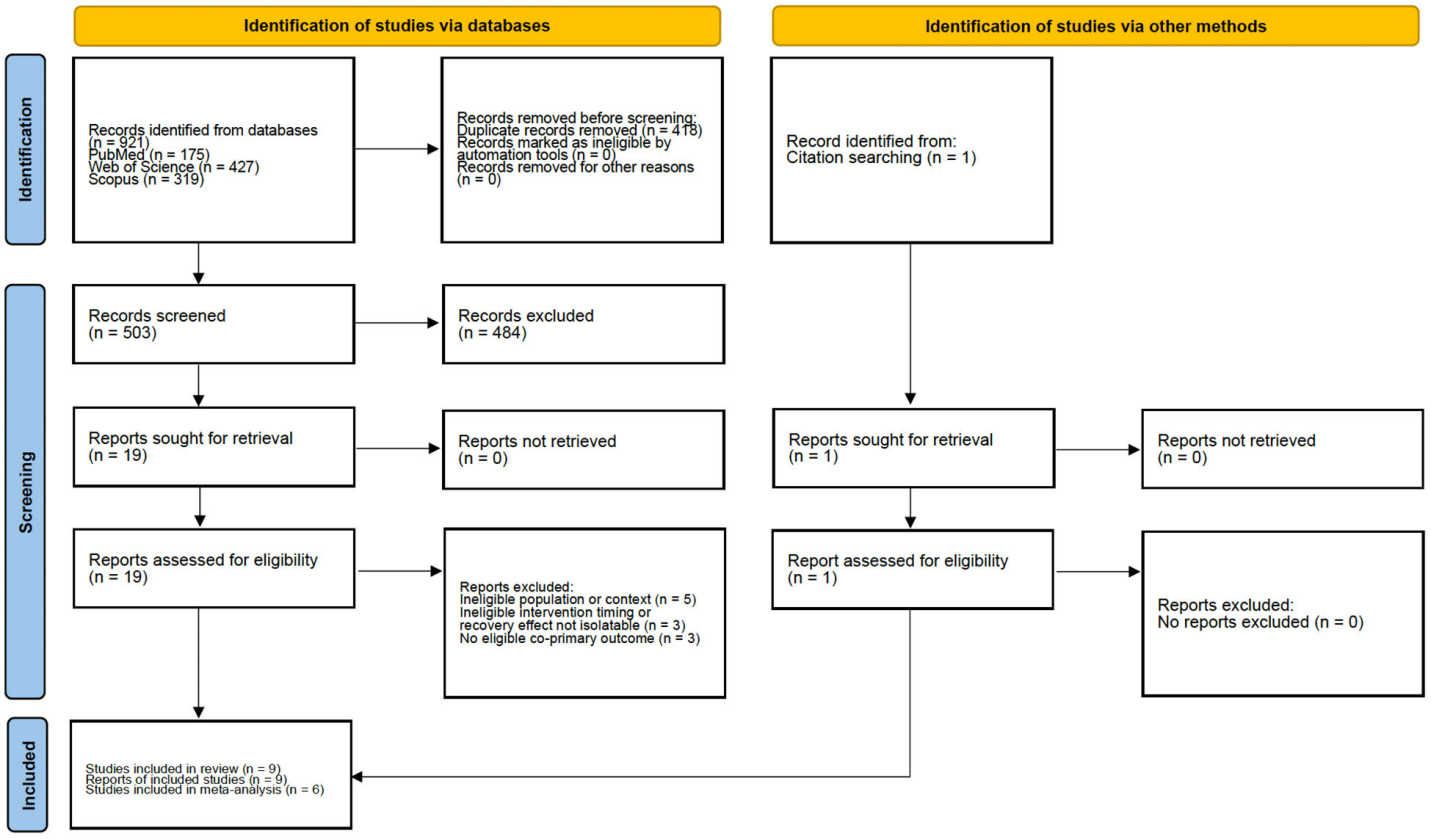
PRISMA 2020 flow diagram of study selection.

The study selection process is summarized in **Figure 1**. Searches identified 921 database records: 175 from PubMed, 427 from Web of Science, and 319 from Scopus. After removal of 418 duplicate records, 503 unique records were screened at title-and-abstract level. Of these, 484 records were excluded, and 19 database-derived reports were sought for retrieval and assessed for eligibility. One additional report was identified through citation searching, retrieved, and assessed. Therefore, 20 full-text reports were assessed for eligibility.

Eleven full-text reports were excluded after detailed assessment. Three reports were excluded because the intervention timing was ineligible or because the post-exercise recovery effect could not be isolated: Allaert et al. (2011) and Geldenhuys et al. (2019) evaluated compression worn during the initial race or training exposure only, with no eligible post-exercise compression recovery phase; Ménétrier et al. (2011) evaluated compression worn during exercise, recovery, and the subsequent performance test, so the independent post-exercise recovery effect could not be separated. Five reports were excluded because the population or context did not meet the review criteria: Ferguson et al. (2014) studied intermittent/team-sport athletes rather than endurance athletes or endurance-trained adults; Govus et al. (2018) included a mixed adult/adolescent sample, with three participants aged under 18 years and no separable adult-only compression-versus-control data; Mizuno et al. (2016) studied physically active adult men with general sport experience rather than endurance athletes or endurance-trained adults; McDonnell et al. (2019) studied regularly active adults and excluded well-trained participants; and Lee et al. (2021) studied healthy physically active young men without a separable endurance-athlete or endurance-trained subgroup. Three reports were excluded because they did not report an eligible co-primary outcome: Kumstát et al. (2019) assessed immediate repeated high-intensity anaerobic cycling performance and metabolic recovery rather than subsequent endurance performance or delayed-onset/perceived muscle soreness; Ménétrier et al. (2014) reported countermovement jump, pressure-pain threshold, palpation-based muscle tension, and limb circumference rather than subsequent endurance performance or self-reported soreness; and Struhár et al. (2018) did not report subsequent endurance performance after recovery or self-reported delayed-onset/perceived muscle soreness. Nine studies were included in the review, and six studies contributed to at least one quantitative synthesis.

### 3.2 Characteristics of included studies

**Table 2** summarizes the main characteristics of the included studies. The nine included studies collectively included 158 analyzed adult participants. Six studies used randomized crossover designs and three used randomized parallel-group designs. The evidence base was geographically concentrated in Europe and Oceania. The participant samples included adult runners, trail or ultra-trail runners, cyclists, and multisport endurance athletes. Sample sizes were small, ranging from 10 to 33 analyzed participants. In a study (Armstrong et al., 2015), 59 participants consented to take part and were randomized, but 33 were analyzed, which was retained as an attrition concern in the risk-of-bias assessment.

**Table 2:** Characteristics of included studies.

Study	Country and region	Design	Sample analyzed	Participants	Comparator	Follow-up
Armstrong et al (Armstrong et al., 2015)	Australia; Oceania	Randomized controlled double-blinded parallel-group trial	33 analyzed; compression n = 16; placebo n = 17	Moderately trained adult marathon runners; overall training volume 6.2 ± 3.2 weekly hours; years running 7.7 ± 7.6; mean marathon time 3:58 ± 0:26 hours:min.	Noncompressive placebo socks	Outcome assessed 14 days after marathon
Bieuzen et al (Bieuzen et al., 2014)	France; Europe	Randomized crossover trial with three conditions	11 analyzed in each crossover condition	Highly trained adult male off-road/trail runners; body mass 72.3 ± 6.8 kg; maximal oxygen uptake 60.1 ± 6.5 mL·min−1·kg−1; training volume 40 to 100 km/week in the preceding 3 months, mean 60 ± 20 km/week.	No-compression control condition	Outcomes assessed at 1, 24, and 48 hours after simulated trail race
Brophy-Williams et al (Brophy-Williams et al., 2017)	Australia; Oceania	Randomized counterbalanced crossover trial	12	Twelve well-trained adult male runners; 5-km run time 19:29 ± 1:18 min:s; height 181.4 ± 6.9 cm; body mass 77.8 ± 6.5 kg; age 30.5 ± 8.1 years; at least four training runs per week for the previous three years; all had prior compression-garment experience.	No compression socks during passive seated recovery	Immediate post-recovery assessment after 60-minute recovery interval
Driller et al (Driller and Halson, 2013)	Australia; Oceania	Randomized crossover trial	10	Ten highly trained cyclists racing at State A or B grade level; age 31 ± 6 years; height 181 ± 6 cm; body mass 75.9 ± 5.9 kg; peak oxygen uptake 66.6 ± 3.8 mL·kg−1·min−1.	Loose-fitting running shorts during passive seated recovery	Immediate post-recovery assessment in second cycling exercise bout after 60-minute recovery interval
de Glanville et al (de Glanville and Hamlin, 2012)	New Zealand; Oceania	Randomized single-blind crossover trial	14	Fourteen trained adult male multisport athletes; age 33.8 ± 6.8 years; body mass 74.6 ± 4.4 kg; height 1.8 ± 0.1 m; mean weekly training duration 6.5 ± 3.4 hours; baseline 40-km mean power output 254.4 ± 18.5 W; 40-km time 66:11 ± 2:10 min:s.	Similar-looking noncompressive placebo garment	Outcome assessed after 24-hour recovery period; garment removed before second time trial
Hill et al (Hill et al., 2014a)	United Kingdom; Europe	Randomized matched parallel-group trial with sham comparator	24; compression n = 12; sham ultrasound n = 12	Twenty-four recreational marathon runners; overall 17 male and 7 female. Compression group: age 47.7 ± 10.8 years, finish time 03:46:45 ± 00:22:30; sham ultrasound group: age 41.1 ± 10.5 years, finish time 03:39:27 ± 00:33:10.	Sham ultrasound non-compression recovery comparator	Outcomes assessed within 1 hour and at 24, 48, and 72 hours after marathon
Li et al (Li et al., 2026)	Netherlands; Europe	Randomized counterbalanced within-subject crossover trial with pseudo-random first allocation and alternating subsequent allocation	12	Recreational adult runners; final analyzed sample had mean age 26 ± 3 years, body mass 71.1 ± 14.7 kg, height 173.8 ± 12.1 cm, maximal oxygen uptake 52.1 ± 9.6 mL·kg−1·min−1, and training volume 394 ± 175 min/week.	No compression/usual noncompressive clothing	24-hour recovery between high-intensity running sessions
Martínez‐Navarro et al (Martínez‐Navarro et al., 2021)	Spain; Europe	Randomized parallel-group controlled trial after race completion	32	Recreational adult ultra-endurance athletes who completed a 107.4-km ultra-trail; whole sample mean age 41 ± 6 years, body mass index 22.8 ± 2.0 kg/m^2^, peak oxygen uptake 54.1 ± 5.2 mL·kg−1·min−1.	No compression garment	48 hours for biochemical markers; 24 hours for delayed-onset muscle soreness
Williams et al (Williams et al., 2021)	United Kingdom; Europe	Single-blind randomized crossover trial with three experimental conditions	10	Trained university-level male cyclists; mean age 21 ± 2 years, height 1.78 ± 0.05 m, body mass 71 ± 11 kg, maximal oxygen uptake 55 ± 10 mL·kg−1·min−1.	Loose-fitting gym clothing; also low-pressure compression comparator	24-hour recovery; garment remained on during day-2 8-km time trial

**Table 3** presents the intervention and comparator characteristics. Compression coverage and dose varied substantially across studies. Three studies evaluated below-knee socks or calf stockings, four evaluated lower-body tights or pants, one evaluated thigh-high stockings, and one evaluated a full-body compression garment. Wear duration ranged from 60 minutes between repeated laboratory exercise bouts to 72 hours after marathon running. Comparator conditions included no compression, usual or noncompressive clothing, loose-fitting clothing, sham ultrasound, and noncompressive placebo garments. Two included studies used noncompressive garment-type placebo comparators, but these studies contributed performance outcomes rather than soreness outcomes to the quantitative syntheses. Within the soreness syntheses, comparator credibility varied: since some studies (Driller and Halson, 2013; Bieuzen et al., 2014; Brophy-Williams et al., 2017; Martínez‐Navarro et al., 2021; Li et al., 2026) used no-compression, usual-clothing, or loose-clothing comparators, whereas a study (Hill et al., 2014a) used a sham-ultrasound comparator rather than a garment placebo.

**Table 3:** Compression-garment and comparator characteristics.

Study	Compression intervention	Body coverage	Applied pressure	Wear duration	Comparator
Armstrong et al (Armstrong et al., 2015)	Below-knee medical-grade compression socks worn continuously for 48 hours after marathon running	Below-knee; ankle to calf/lower leg	30–40 mmHg at ankle; 21–28 mmHg at calf	48 hours continuously after marathon	Noncompressive SmartKnit seamless knee-high diabetic socks
Bieuzen et al (Bieuzen et al., 2014)	Elastic calf compression stockings applied during recovery after simulated trail running	Both calves; below knee to lower leg/calf	Constant pressure of 20 mmHg	Exact total wear duration unclear from report; recovery condition included post-run follow-up at 1, 24, and 48 hours	Run and recovery without compression stockings (no-compression condition control)
Brophy-Williams et al (Brophy-Williams et al., 2017)	Commercial below-knee compression socks worn during short-term post-exercise recovery	Lower leg and foot; below-knee sports compression socks	23 ± 11 mmHg at maximal calf girth, 22 ± 8 mmHg at upper ankle, and 19 ± 8 mmHg at lower ankle	60 minutes	No compression socks during the matched 60-minute passive seated recovery period
Driller et al (Driller and Halson, 2013)	Full-length lower-body compression tights worn during short-term post-exercise recovery	From the superior aspect of the medial malleolus of the ankle to slightly superior to the iliac crest	Approximately 20.5 ± 3.1 mmHg at the calf decreasing to 11.8 ± 2.6 mmHg at the mid-thigh; not measured in the current study participants	60 minutes	Loose-fitting running shorts during the same 60-minute passive recovery period
de Glanville et al (de Glanville and Hamlin, 2012)	Full-leg graduated compression garment worn continuously during 24-hour recovery after a 40-km cycling time trial	Full lower body from ankle to hip/waist	6.0 ± 2.4 mmHg at 10 cm above the sphyrion; 14.7 ± 2.5 mmHg at midcalf; 11.8 ± 2.5 mmHg at midtrochanterion–tibiale laterale	24 hours continuously, with brief removal permitted for showering if needed	Similar-looking noncompressive placebo garment
Hill et al (Hill et al., 2014a)	Lower-limb compression tights worn continuously for 72 hours after marathon running	Lower limbs; tights fitted to male and female participants	Thigh: 9.9 ± 2.0 mmHg standing and 17.5 ± 3.5 mmHg during muscle contraction; calf: 19.3 ± 2.6 mmHg standing and 24.4 ± 5.0 mmHg during muscle contraction	72 hours continuously after marathon completion, removed only to shower	Single 15-minute sham ultrasound treatment applied to quadriceps, hamstrings, and gastrocnemius within 1 hour after marathon completion
Li et al (Li et al., 2026)	Medical thigh-high compression stockings worn during post-exercise recovery	Thigh-high, open-toe stockings; lower limbs from foot/ankle to upper thigh	Manufacturer-estimated 23–32 mmHg	Recorded total wear duration 12 h 37 min ± 59 min within a 24-hour recovery interval; excluded sleep or bathing	Usual non-compressive clothing/no stockings in control condition
Martínez‐Navarro et al (Martínez‐Navarro et al., 2021)	Full-body compression garment worn immediately after ultra-trail race	Full body garment	10–15 mmHg according to manufacturer specification and AITEX validation	First 24 hours following 107.4-km ultra-trail race, including night sleep	No compression garment for immediate 24 h following race
Williams et al (Williams et al., 2021)	High-pressure lower-limb compression garment worn during recovery and subsequent cycling time trial	Lower limbs/pelvis; pressure measured at distal hem, calf, mid-thigh, head of femur, and posterior superior iliac spine	Measured pressures 11 ± 3, 15 ± 3, 10 ± 3, 8 ± 2, and 6 ± 1 mmHg at distal hem, maximal calf, mid-thigh, head of femur, and posterior superior iliac spine	Approximately 24 hours; garment not removed throughout the recovery period and was worn during the 8-km time trial	Loose-fitting gym clothing control; also compared with low-pressure compression garment
Williams et al (Williams et al., 2021)	Low-pressure lower-limb compression garment worn during recovery and subsequent cycling time trial	Lower limbs/pelvis; pressure measured at distal hem, calf, mid-thigh, head of femur, and posterior superior iliac spine	Measured pressures 7 ± 3, 7 ± 3, 5 ± 2, 5 ± 2, and 5 ± 1 mmHg at distal hem, maximal calf, mid-thigh, head of femur, and posterior superior iliac spine	Approximately 24 hours; garment not removed throughout the recovery period and was worn during the 8-km time trial	Loose-fitting gym clothing control; also compared with high-pressure compression garment

The included studies reported one or both co-primary outcome domains. Subsequent endurance performance was assessed using standardized treadmill time to exhaustion, 5-kilometre running time trials, repeated cycling performance, 40-kilometre cycling time trials, or 8-kilometre cycling time-trial performance. Delayed-onset or perceived muscle soreness was assessed using visual analogue scales or numerical soreness scales, typically during a movement-based task such as a squat or half-squat. The primary timing window was approximately 24 hours post-exercise, defined as 20 to 30 hours after the exercise stimulus. Additional extracted windows included immediate to 2 hours, greater than 30 to 48 hours, greater than 48 to 72 hours, and greater than 72 hours post-exercise. The immediate-to-2-hour window is reported below as acute post-exercise perceived soreness or discomfort rather than classic delayed-onset muscle soreness, because these measurements were obtained approximately 1 hour after exercise or after a 60-minute recovery interval.

Garment provenance and manufacturer or sponsor involvement were incompletely reported across the included studies. Bieuzen et al. (2014) reported that the study was funded by Sigvaris Sports, which provided the compression stockings used in the experiment. The authors stated that the funder had no role in study design, data collection and analysis, publication decision, or manuscript preparation. Brophy-Williams et al. (2017) acknowledged 2XU for supplying the compression garments and stated that 2XU had no input into study design, data collection or analysis, or publication. Driller and Halson (2013) reported that 2XU, the manufacturer of the compression garments, had a partnership with the Australian Institute of Sport, but that the project was funded by the Australian Institute of Sport and conducted independently with no 2XU input into design, data collection, analysis, or reporting. Martínez‐Navarro et al. (2021) reported collaboration with AITEX, the Textile Research Institute, which provided the full-body compression garments, and stated that sponsors had no involvement in study design, data collection, analysis and interpretation, manuscript writing, or submission. For Armstrong et al. (2015); de Glanville and Hamlin (2012); Hill et al. (2014a); Li et al. (2026), and Williams et al. (2021), the included full texts did not clearly report manufacturer provision, manufacturer funding, or supplier involvement for the compression garments.

### 3.3 Risk of bias in included studies

Risk of bias was assessed using RoB 2 at the outcome-result level. Eleven outcome-level assessments were conducted. Ten outcome results were judged to be at high risk of bias, and one was judged as having some concerns. These assessments comprised five subsequent endurance-performance results and six soreness results. The performance assessments were Armstrong et al. (2015); Brophy-Williams et al. (2017); Driller and Halson (2013); de Glanville and Hamlin (2012), and Williams et al. (2021). The soreness assessments were Bieuzen et al. (2014); Brophy-Williams et al. (2017); Driller and Halson (2013); Hill et al. (2014a); Li et al. (2026), and Martínez‐Navarro et al. (2021). Williams et al. (2021) was included in the narrative performance synthesis and in the certainty assessment for subsequent endurance performance, but as mixed recovery-plus-performance-wear evidence rather than as clean recovery-only evidence. It was judged to be at high risk of bias for the performance outcome and was treated as indirect for the recovery-only question because the garment was worn during both the recovery period and the subsequent 8-km cycling time trial.

Across all outcome results, the randomization process, deviations from intended interventions, and selection of the reported result were consistently rated as some concerns, primarily because of incomplete reporting of sequence generation, allocation concealment, blinding, adherence, or prespecified analysis plans. Missing outcome data were generally rated as low risk, except in Armstrong et al. (2015) and Li et al. (2026). In Armstrong et al. (2015), 59 participants consented to take part and were randomized, but only 33 provided complete pre- and post-marathon treadmill data for analysis. In Li et al. (2026), 17 participants entered the study and 12 completed the crossover protocol. These issues were considered in the missing outcome data domain and in the certainty assessment.

**Figure 2:**
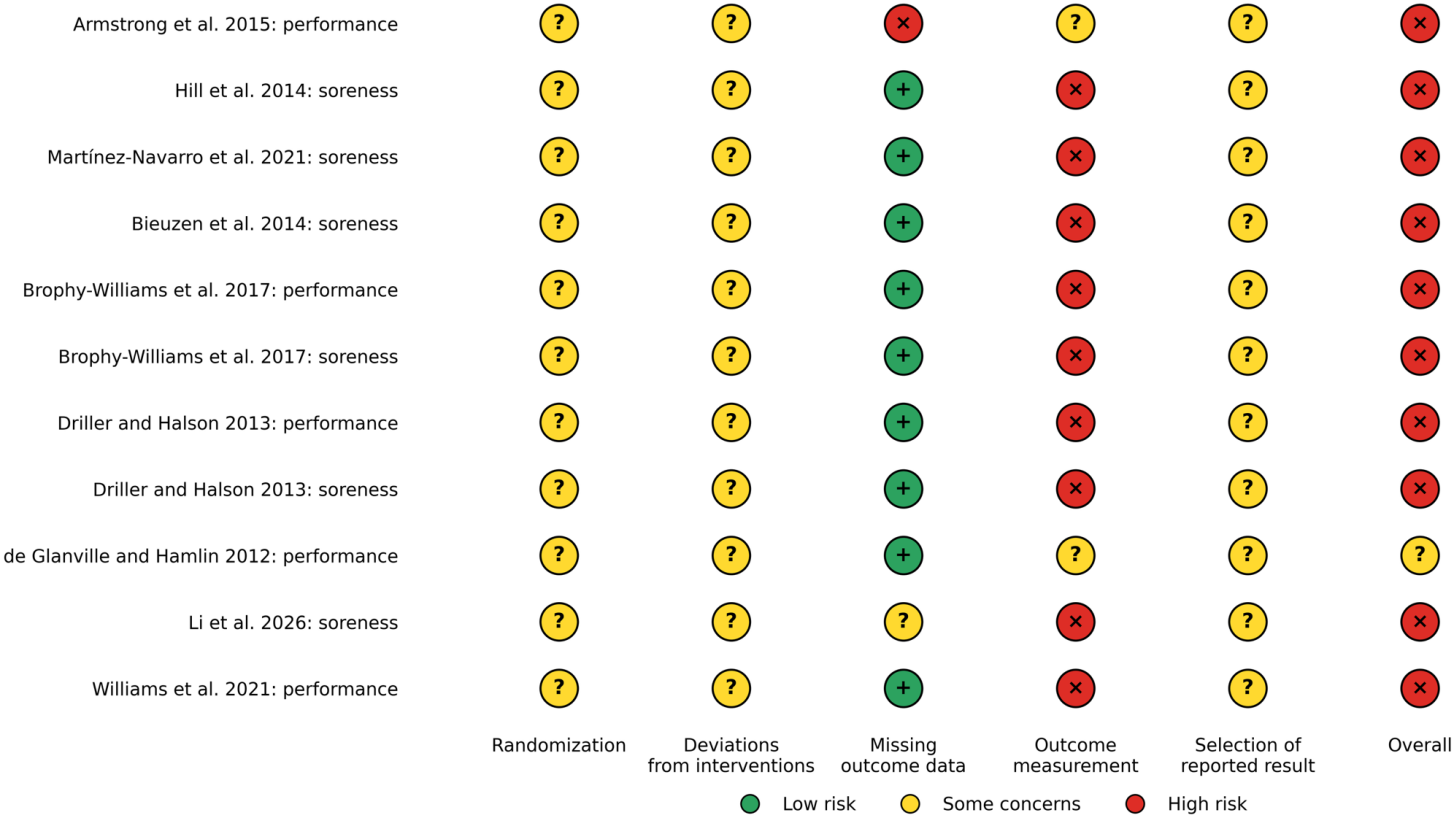
Risk-of-bias traffic-light plot by outcome result. Green circles indicate low risk, yellow circles indicate some concerns, and red circles indicate high risk.

**Figure 3:**
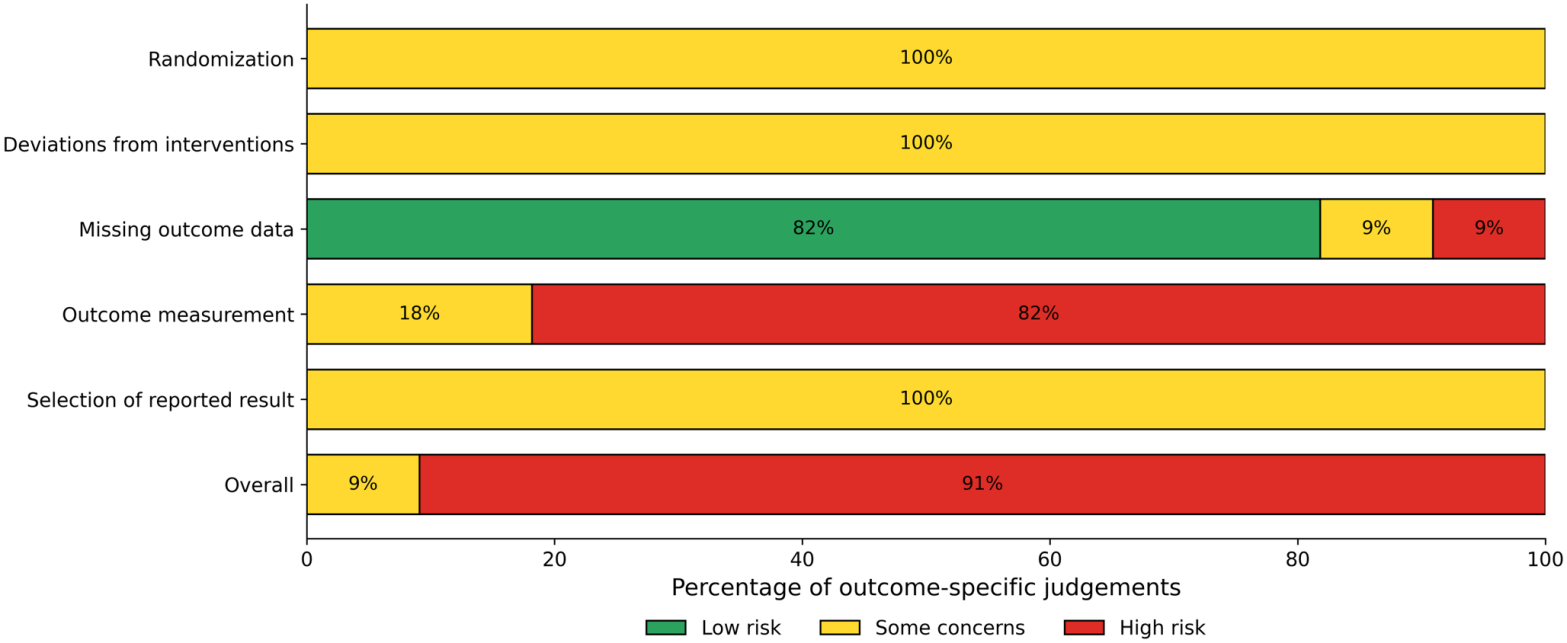
Summary of risk-of-bias judgements across domains. Bars show the percentage of outcome-specific judgements in each risk-of-bias category.

Outcome measurement was the main high-risk domain, particularly for subjective soreness outcomes assessed under unblinded or poorly blinded conditions and for performance outcomes requiring maximal effort under distinguishable recovery conditions. This issue was especially important for the soreness syntheses because perceived soreness was an athlete-reported subjective outcome and most soreness comparisons used no-compression, usual-clothing, loose-clothing, or non-garment sham comparators rather than sensorially equivalent placebo garments. In Hill et al. (2014a), the compression and sham-ultrasound groups also differed in mean age despite similar marathon finishing times (compression 47.7 ± 10.8 years; sham ultrasound 41.1 ± 10.5 years), and this possible baseline imbalance was considered when judging the randomization/baseline domain and interpreting the subjective soreness result. The domain judgements are shown in **Figures 2** and **3**.

### 3.4 Results of individual studies

**Table 4** summarizes the main eligible results from each included study, including the soreness scale range, extracted time point, effect direction, and key interpretive notes. For subsequent endurance performance, four studies provided recovery-only or largely recovery-focused contrasts. Armstrong et al. (2015) reported a statistically significant improvement in treadmill time to exhaustion at 14 days after marathon running in the compression group compared with the noncompressive placebo-sock group. The compression group improved by 52.4 ± 103.3 seconds, whereas the placebo group decreased by 61.7 ± 129.6 seconds (p = 0.0091), although interpretation is limited by high attrition between randomization and analysis. Brophy-Williams et al. (2017) reported a smaller performance decrement between repeated 5-kilometre treadmill time trials after compression-sock recovery than after no compression, but the between-condition difference was not statistically significant. Driller and Halson (2013) reported better maintenance of cycling performance after a 60-minute recovery period with lower-body compression garments than with loose-fitting shorts. de Glanville and Hamlin (2012) reported improved second 40-kilometre cycling time-trial performance after 24 hours of recovery in a graduated compression garment compared with a similar-looking noncompressive placebo garment; importantly, the garments were removed before the second time trial, allowing a recovery-focused interpretation. One additional performance study was retained only as mixed-timing evidence. Williams et al. (2021) reported improved day-2 8-kilometre cycling time-trial performance with high-pressure compression, however, the garment was worn during both the recovery period and the subsequent performance test.

**Table 4:** Main results of individual included studies.

Study	Main comparison	Main results	Main finding
Armstrong et al (Armstrong et al., 2015)	Below-knee compression socks for 48 hours after marathon running versus noncompressive placebo socks.	Change from pre-marathon to 14 days post-marathon: compression +52.4 ± 103.3 s, n = 16; placebo -61.7 ± 129.6 s, n = 17; p = 0.0091. Absolute time to exhaustion: compression 1765 ± 254 to 1811 ± 319 s; placebo 1839 ± 382 to 1777 ± 386 s.	Reported result favors compression for functional endurance recovery.
Bieuzen et al (Bieuzen et al., 2014)	Sigvaris Recovery calf compression stockings during post-run recovery versus no compression during run or recovery.	1 h: recovery compression 5.0 ± 1.9 vs no compression 4.8 ± 2.6. 24 h: recovery compression 4.2 ± 1.9 vs no compression 4.1 ± 1.9. Meta-analysis coding: g = -0.08 at 1 h and g = -0.05 at 24 h, because positive values favor lower soreness with compression.	Recovery-only compression did not show a clear soreness benefit. The broader study benefit concerned compression worn during running, not recovery-only compression.
Brophy-Williams et al (Brophy-Williams et al., 2017)	Compression socks during 60-minute recovery after the first 5-km treadmill time trial versus no compression during the same recovery interval.	Performance decrement: compression +5.3 ± 20.7 s vs control +15.9 ± 13.3 s; between-condition difference 10.6 ± 14.5 s favoring compression, p = 0.14. Soreness: compression 4.8 ± derived SD 2.05 pre-recovery to 2.8 ± derived SD 1.10 post-recovery; control 3.8 ± derived SD 1.89 to 4.0 ± derived SD 2.05; reported change difference -2.3 ± 1.5 AU, p < 0.01.	Compression reduced short-term perceived soreness, but pre-recovery soreness was higher in compression than control. Post-recovery values were used in the immediate-to-2-hour synthesis.
Driller et al (Driller and Halson, 2013)	Lower-body compression tights during 60-minute passive recovery after the first cycling bout versus loose-fitting running shorts.	Performance: LBCG 312.1 ± 23.3 W in bout 1 and 311.5 ± 25.5 W in bout 2; control 314.9 ± 28.8 W and 308.1 ± 24.4 W; reported effect 1.8 ± 1.3% favoring compression, p < 0.05. Soreness: LBCG 7.4 ± 1.9 pre-recovery to 1.9 ± 1.5 post-recovery; control 6.5 ± 2.4 to 3.2 ± 2.5; reported change difference -1.1 ± 1.2 AU; likelihood 81/17/2.	Compression improved maintenance of cycling performance and showed a non-significant direction toward reduced perceived soreness. No placebo/blinding.
de Glanville et al (de Glanville and Hamlin, 2012)	Full-leg graduated compression garment worn continuously for 24 hours after a 40-km cycling time trial versus similar-looking noncompressive placebo garment.	Mean performance time improved by -1.2 ± 0.4% with compression versus placebo; average power output 3.3 ± 1.1% higher; 90% confidence limits reported. Oxygen cost and RPE differences trivial or unclear.	Compression favored subsequent endurance-performance recovery after 24 hours. Placebo-controlled single-blind crossover design strengthens interpretation.
Hill et al (Hill et al., 2014a)	Lower-limb compression tights worn continuously for 72 hours after marathon running versus 15-minute sham ultrasound comparator.	Soreness: time-by-group effect reported as F(4,1) = 30.8, p < 0.001; group effect F(1,22) = 4.451, p = 0.046. At 24 h, sham soreness increased from 26.8 to 36.4 mm, while compression soreness decreased from 31.0 to 13.9 mm; *post hoc* p < 0.05. No significant group effects for MVIC, CK, or C-RP.	Compression improved subjective soreness at 24 hours but not objective muscle-function or biomarker outcomes. Hill et al. reported the perceived-soreness time × group effect as F(4,1) = 30.8, p < 0.001, and the group effect as F(1,22) = 4.451, p = 0.046. Based on the stated two-group design with 24 participants and five repeated time points, the uncorrected degrees of freedom expected for the time × group interaction would be F(4,88). No corrected value or erratum was identified.
Li et al (Li et al., 2026)	Medical thigh-high compression stockings during the post-exercise recovery period versus no compression/usual non-compressive clothing in a crossover design.	Day 1 Post: compression 58.17 ± 26.01 mm vs control 51.00 ± 20.60 mm. Day 2 Pre: compression 38.83 ± 21.00 mm vs control 46.25 ± 25.06 mm. DOMS declined from Day 1 Post to Day 2 Pre in compression (Z = -2.904, p = 0.004) but not control (Z = -1.179, p = 0.239).	Objective markers were not improved, but perceived recovery may have improved. Direction differs by time point; Day 2 Pre was selected for the approximately-24-hour synthesis.
Martínez‐Navarro et al (Martínez‐Navarro et al., 2021)	Full-body compression garment for 24 h immediately after a 107.4-km ultra-trail race versus no compression garment.	Corrected posterior-leg DOMS relative change at 24 h: full-body compression 11.0 ± 46.2% vs control 112.3 ± 170.4%; reported p = 0.03 and Cohen d = 0.8. Corrected meta-analysis coding: Hedges g = 0.79, 95% CI 0.07 to 1.51.	Possibly favorable to reduce muscle soreness but not muscle damage and inflammatory response
Williams et al (Williams et al., 2021)	High-pressure and low-pressure lower-limb compression garments during a 24-h period after high-intensity cycling versus loose-fitting gym clothing; garments remained on during day-2 8-km time trial.	8-km mean work rate: high pressure 277 ± 83 W, control 266 ± 89 W, low pressure 265 ± 77 W; p < 0.05 for high pressure versus control/low pressure. Subjective soreness averaged approximately 2 ± 1 cm irrespective of condition; all p > 0.05.	High-pressure compression improved day-2 cycling performance, but the recovery-only effect was not isolatable because garments were worn during the subsequent performance test. Soreness was minimal and not different between conditions.

For delayed-onset or perceived muscle soreness, Bieuzen et al. (2014) did not show a clear benefit of compression stockings worn during recovery after simulated trail running. For the recovery-compression versus no-compression contrast, soreness values were slightly higher with compression at 1 hour (5.0 ± 1.9 vs 4.8 ± 2.6 on a 10-cm visual analogue scale) and at 24 hours (4.2 ± 1.9 vs 4.1 ± 1.9). The clearer benefit in that study concerned compression worn during running, not compression worn only during recovery. Brophy-Williams et al. (2017) reported a statistically significant reduction in perceived muscle soreness during a 60-minute recovery interval with compression socks compared with no compression, however, pre-recovery soreness was higher in the compression condition than in control (4.8 vs 3.8 arbitrary units), so the short-term soreness result should be interpreted with this pre-recovery imbalance in mind. Driller and Halson (2013) reported lower post-recovery perceived soreness after lower-body compression garments than after loose-fitting shorts, although the study reported this as a non-significant moderate effect.

Hill et al. (2014a) reported significantly lower perceived soreness at 24 hours after marathon running in the compression group compared with a sham-ultrasound comparator. Li et al. (2026) reported that soreness declined from immediately after the first high-intensity running session to the next-day pre-exercise measurement in the compression condition but not in the control condition. Direction depended on time point since on Day 1 post-exercise values were numerically higher with compression than control (58.17 ± 26.01 mm vs 51.00 ± 20.60 mm), whereas the Day 2 pre-exercise value selected for the approximately-24-hour synthesis was lower with compression (38.83 ± 21.00 mm vs 46.25 ± 25.06 mm). Martínez‐Navarro et al. (2021) reported a smaller posterior-leg soreness change at 24 hours after a 107.4-kilometre ultra-trail race with full-body compression than with no compression.

Adherence, tolerability, thermal discomfort, and adverse events were inconsistently reported. Armstrong et al. (2015) provided the clearest adverse-event reporting, namely one study participant was unable to wear the compression socks for the full 48-hour post-marathon period because of subjective discomfort attributed to the compressive effect; symptoms resolved within 30 minutes after sock removal, no long-term consequences were reported, and no other participants in either group reported adverse events. Armstrong et al. (2015) also noted that below-knee socks were chosen partly to improve compliance because above-knee compression socks may have poorer user compliance due to heat retention and discomfort. Li et al. (2026) reported that all included participants confirmed adherence to the compression-stocking protocol and that included participants did not report injuries during the experiment, but five participants dropped out before completing one block because of scheduling conflicts, sickness, or injuries, and early termination of some running protocols was attributed to exhaustion or stomach discomfort rather than to the compression stockings. Williams et al. (2021) explicitly assessed subjective discomfort and additional questions on garment comfort, irritability, and fit; subjective sleep quality, fatigue, and discomfort did not differ significantly between conditions, although the authors also acknowledged that adherence during the 24-hour recovery period could not be confirmed with complete certainty. de Glanville and Hamlin (2012) did not report garment-related adverse events, but they did report a large drop in systolic, diastolic, and mean blood pressure immediately after removal of the compression garment following 24 hours of wear; no associated clinical symptoms or adverse events were reported. Martínez‐Navarro et al. (2021) did not report observed adverse-event counts. Bieuzen et al. (2014); Brophy-Williams et al. (2017); Driller and Halson (2013), and Hill et al. (2014a) did not provide explicit adverse-event or tolerability reporting beyond protocol descriptions of garment use. Therefore, absence of reported adverse events should not be interpreted as evidence that post-exercise compression garments are free of discomfort, adherence problems, or harms, particularly when garments are worn for prolonged periods such as 24 to 72 hours.

### 3.5 Results of quantitative synthesis

**Figure 4:**
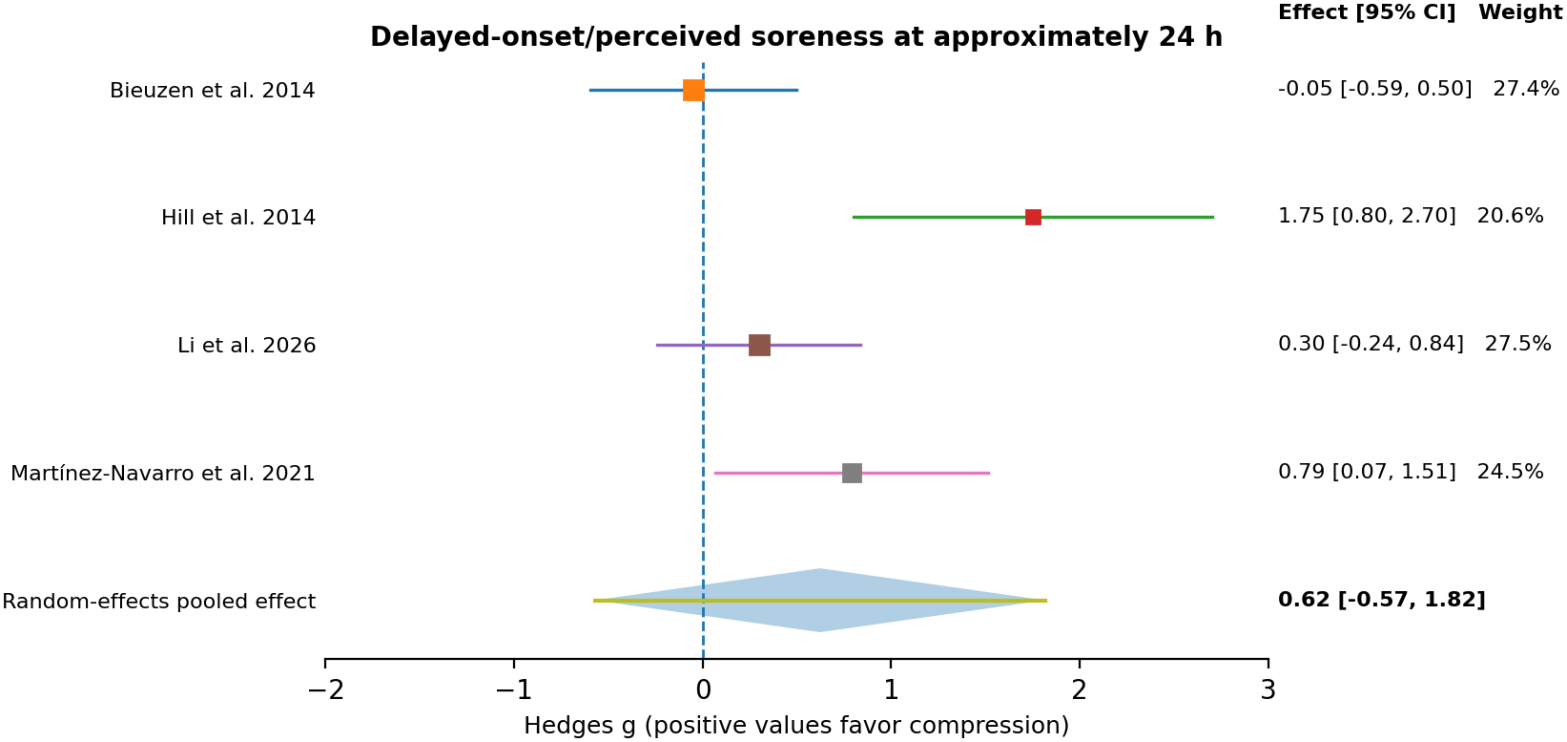
Exploratory random-effects meta-analysis of delayed-onset or perceived muscle soreness at approximately 24 hours. Positive Hedges g values indicate lower soreness with compression. Random-effects REML/HKSJ estimate: g = 0.62, 95% CI -0.57 to 1.82; tau-squared = 0.399; I-squared = 74.0%.

**Figure 5:**
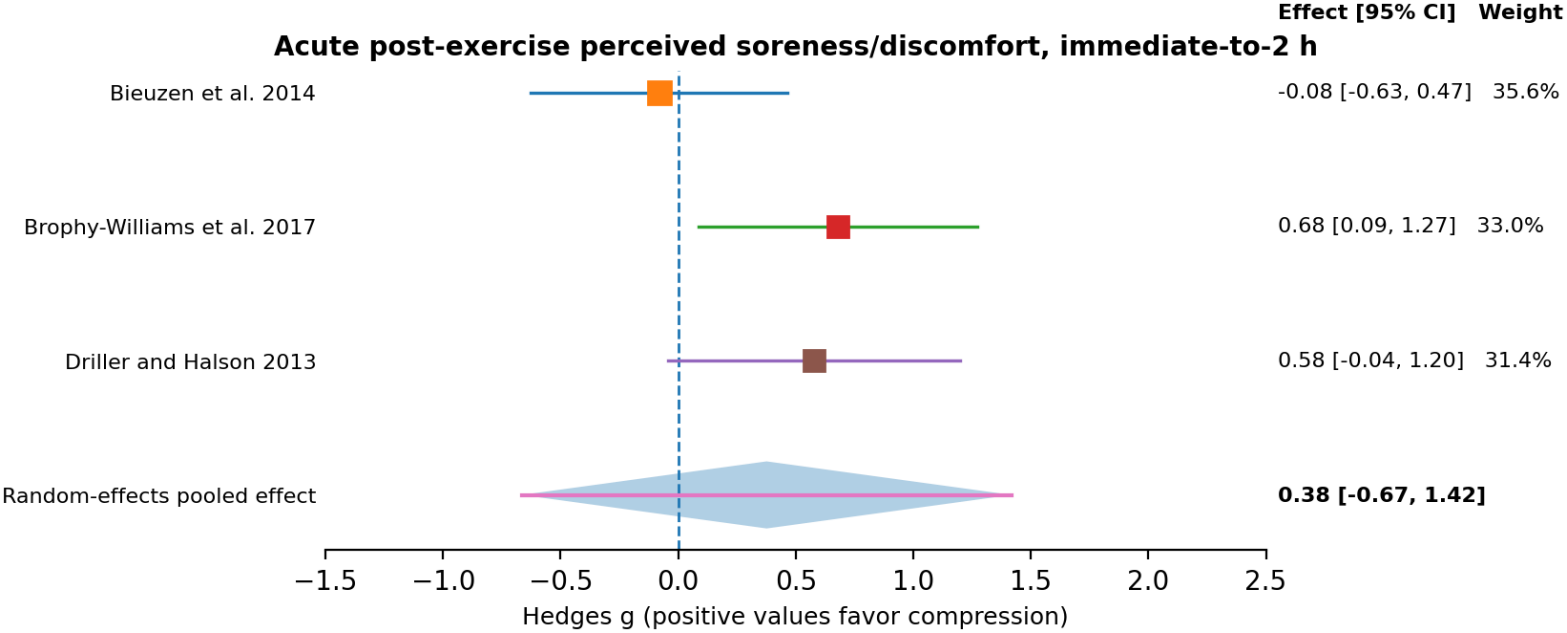
Exploratory random-effects meta-analysis of acute post-exercise perceived soreness/discomfort in the immediate-to-2-hour window. Positive Hedges g values indicate lower soreness with compression. Random-effects REML/HKSJ estimate: g = 0.38, 95% CI -0.67 to 1.42; tau-squared = 0.093; I-squared = 51.0%.

Two exploratory meta-analyses were conducted because at least three studies reported soreness outcomes within the same prespecified time window (**Figures 4**, **5**). Both analyses synthesized soreness using standardized mean differences corrected for small-sample bias. Positive Hedges g values indicate lower soreness with compression. Random-effects models were estimated using restricted maximum likelihood, with Hartung-Knapp-Sidik-Jonkman inference. Pooling was retained only as an exploratory summary because the studies differed in exercise stimulus, comparator type, credibility of blinding, anatomical site or task used to elicit soreness, scale range, study design, and recovery protocol. The minimum basis for exploratory pooling was that all included effects assessed post-exercise compression in adult endurance athletes, reported a self-reported soreness outcome within the same prespecified time window, and could be aligned so that one independent effect per study contributed to a standardized direction-coded estimate. The pooled estimates should therefore be interpreted alongside the individual studies analysis rather than as definitive clinically exchangeable effects. The exact time point, data type, derivation basis, direction coding, standard error, variance, and assumed crossover correlation for each included effect are provided in **Supplementary Material**.

For delayed-onset or perceived muscle soreness at approximately 24 hours, four studies contributed to the primary time-windowed exploratory meta-analysis. The pooled effect favored compression but remained statistically uncertain (Hedges g = 0.62, 95% confidence interval -0.57 to 1.82; p = 0.195). Statistical heterogeneity was substantial and imprecisely estimated (I-squared = 74.0%, approximate 95% confidence interval 20.7% to 89.7%; tau-squared = 0.399; Q = 11.55, p = 0.009). The Q-test p value is reported descriptively using the conventional heterogeneity-screening threshold of 0.10, but it was not used as the sole basis for deciding whether pooling was appropriate because only four studies were available. Bieuzen et al. (2014) contributed an approximately null/slightly unfavorable effect for recovery compression (g = -0.05), Li et al. (2026) contributed a small favorable effect at Day 2 pre-exercise (g = 0.30), Martínez‐Navarro et al. (2021) contributed a moderate-to-large favorable effect (g = 0.79), and Hill et al. (2014a) contributed a large favorable effect (g = 1.75). Comparator credibility varied across these studies since Bieuzen et al. (2014); Li et al. (2026), and Martínez‐Navarro et al. (2021) used no-compression or usual-clothing comparators, whereas Hill et al. (2014a) used sham ultrasound rather than a garment placebo. A fixed-effect sensitivity analysis gave Hedges g = 0.44 (95% confidence interval 0.12 to 0.76; p = 0.007), indicating that nominal statistical inference depended on model choice. Sensitivity analyses using assumed crossover correlations of r = 0.25 and r = 0.75 did not change the random-effects conclusion that the pooled estimate was imprecise and exploratory.

For clinical interpretation, the corrected 24-hour pooled SMD was back-converted using the pooled Day 2 pre-exercise standard deviation from the Li et al. (2026). DOMS VAS data selected for synthesis (23.1 mm on a 0–100 mm scale). This corresponds to an illustrative mean difference of approximately 14 mm lower soreness with compression, with a 95% confidence interval ranging from approximately 13 mm higher soreness to 42 mm lower soreness. This back-conversion is approximate because soreness scales, anatomical sites, elicitation tasks, and reporting formats differed across studies. No DOMS minimal important difference was available from the included endurance-athlete studies. Therefore, a 10-mm change on a 0–100 mm pain VAS was used only as a contextual benchmark (Myles et al., 2017).

### 3.6 Certainty of evidence

The certainty of evidence was assessed using the Grading of Recommendations Assessment, Development and Evaluation framework. **Table 5** presents the outcome-level certainty judgements. The certainty of evidence was rated as very low for subsequent endurance performance and for both synthesized soreness time windows. The main reasons for downgrading were high risk of bias, small sample sizes, wide confidence intervals crossing the null effect, and heterogeneity across exercise protocols, compression doses, comparator types, and measurement timings. Publication bias could not be assessed formally because each synthesis contained fewer than 10 studies.

**Table 5:** Certainty of evidence using the Grading of Recommendations Assessment, Development and Evaluation framework.

Outcome	Evidence base	Effect estimate or synthesis approach	Risk of bias	Inconsistency	Indirectness	Imprecision	Publication bias	Overall certainty	Interpretation
Subsequent endurance performance after post-exercise recovery	5 studies; 79 participants; narrative synthesis only	Not pooled because fewer than three studies were sufficiently comparable within a single prespecified recovery window and one 24-hour study did not isolate recovery-only effects.	Very serious	Serious	Serious	Serious	Not formally assessed; fewer than 10 studies and narrative synthesis only	Very low	Several individual studies favored compression, but certainty is very low because the evidence base was small, heterogeneous, and mostly at high risk of bias.
Delayed-onset or perceived muscle soreness at approximately 24 hours	4 studies; 79 participants	Exploratory random-effects REML/HKSJ: Hedges g = 0.62, 95% CI -0.57 to 1.82; I-squared = 74.0% (approximate 95% CI 20.7% to 89.7%); tau-squared = 0.399. Fixed-effect sensitivity: g = 0.44, 95% CI 0.12 to 0.76.	Very serious	Serious	Not serious	Serious	Not formally assessed; fewer than 10 studies	Very low	The pooled estimate favored compression but the random-effects confidence interval included no effect and potentially meaningful benefit; heterogeneity and comparator credibility limit inference.
Delayed-onset or perceived muscle soreness immediate to 2 hours	3 studies; 33 participants	Exploratory random-effects REML/HKSJ: Hedges g = 0.38, 95% CI -0.67 to 1.42; I-squared = 51.0% (approximate 95% CI 0.0% to 87.8%); tau-squared = 0.093. Fixed-effect sensitivity: g = 0.36, 95% CI 0.02 to 0.70.	Very serious	Serious	Not serious	Serious	Not formally assessed; fewer than 10 studies	Very low	The pooled estimate favored compression, but the window reflects acute perceived soreness/discomfort rather than classic DOMS, and random-effects uncertainty was large.

Downgrade notation: serious = one-level downgrade; very serious = two-level downgrade. Randomized evidence started at high certainty. Subsequent endurance performance was downgraded by at least five levels before capping at very low certainty: risk of bias (-2), inconsistency (-1), indirectness (-1), and imprecision (-1). The 24-hour and immediate-to-2-hour soreness syntheses were each downgraded by at least four levels before capping at very low certainty: risk of bias (-2), inconsistency (-1), and imprecision (-1). Publication bias was not assessable and was not used as an additional downgrade.

## 4 Discussion

This systematic review with meta-analysis evaluated whether static wearable compression garments used after exercise improve subsequent endurance performance and reduce delayed-onset or perceived muscle soreness in adult endurance athletes. The evidence base comprised nine controlled studies, most of which enrolled small samples of runners, trail runners, cyclists, or multisport endurance athletes. The review identified several directionally favorable estimates for perceived soreness, but not a uniformly consistent pattern across studies since some effects were close to null or statistically uncertain, and the overall certainty of evidence was very low. The primary meta-analysis for delayed-onset or perceived muscle soreness at approximately 24 hours produced a point estimate favoring compression, but the confidence interval crossed the null effect, heterogeneity was substantial, and certainty was very low. The immediate-to-2-hour synthesis of acute post-exercise perceived soreness/discomfort also produced a numerically favorable point estimate, but the estimate was imprecise, statistically uncertain, and based on only three studies.

Individual performance studies reported favorable results in some recovery-only or largely recovery-focused contexts, but these findings should not be interpreted as evidence of a consistent endurance-performance effect (de Glanville and Hamlin, 2012; Driller and Halson, 2013; Armstrong et al., 2015; Brophy-Williams et al., 2017). The performance evidence could not be pooled in a main meta-analysis because studies differed markedly in recovery windows, endurance modalities, performance tests, comparator conditions, reporting formats, and whether the effect of post-exercise recovery could be isolated. This distinction is important because the review question focused specifically on recovery use rather than performance while wearing compression. The main contribution of this review is therefore to separate post-exercise compression from in-exercise or mixed-timing compression use and to show that, although subjective recovery may improve in some contexts, the current evidence does not establish a precise or high-certainty effect on either soreness or subsequent endurance performance.

### 4.1 Interpretation of effects on delayed-onset and perceived muscle soreness

The soreness evidence suggests that compression garments may reduce perceived discomfort after endurance exercise, but the magnitude and consistency of the effect remain uncertain. The approximately-24-hour synthesis was driven by studies that differed substantially in exercise stimulus and garment protocol, ranging from laboratory or simulated trail-running designs to marathon and ultra-trail contexts (Bieuzen et al., 2014; Hill et al., 2014a; Martínez‐Navarro et al., 2021; Li et al., 2026). Hill et al (Hill et al., 2014a). reported lower soreness at 24 hours after marathon running in participants allocated to lower-limb compression tights compared with a sham-ultrasound comparator, whereas Bieuzen et al (Bieuzen et al., 2014). did not show a clear soreness benefit for compression stockings worn during recovery after simulated trail running. Li et al (Li et al., 2026). reported a within-condition decline in soreness during the overnight recovery period when compression stockings were used, but between-condition differences at matched time points were less conclusive. Martínez‐Navarro et al (Martínez‐Navarro et al., 2021). reported lower posterior-leg soreness change after ultra-trail running with full-body compression, but the comparison was based on a specific anatomical soreness region and percentage-change values.

The immediate-to-2-hour soreness synthesis also favored compression but remained statistically uncertain. In short-turnaround exercise models, Brophy-Williams et al (Brophy-Williams et al., 2017). reported a significant reduction in perceived soreness over a 60-minute recovery interval with compression socks, whereas Driller and Halson (Driller and Halson, 2013) observed a directionally favorable but non-significant soreness effect after lower-body compression during a 60-minute passive recovery period. These findings indicate that compression may influence short-term perceptual recovery, particularly when soreness is assessed soon after exercise and when the participant can perceive the garment during the recovery interval. However, this same perceptual mechanism complicates interpretation because many comparisons were not credibly blinded. The tendency aligns with broader compression-garment literature suggesting that perceptual outcomes may respond more consistently than objective physiological or performance outcomes (Engel et al., 2016; Brown et al., 2017; Weakley et al., 2022).

Differences in exercise-induced damage may also explain variation across studies. Marathon and ultra-trail running impose prolonged eccentric loading and may produce soreness profiles different from shorter laboratory running bouts or cycling protocols. Conversely, highly trained athletes may experience attenuated soreness responses because of training adaptation and repeated exposure to similar mechanical stress. The included studies also used different soreness instruments, body regions, and assessment tasks, including movement-evoked visual analogue scales, half-squat soreness ratings, and region-specific palpation-based ratings. These methodological differences support the use of standardized mean differences but also limit clinical interpretability because a standardized pooled effect cannot be directly translated into a consistent reduction on a single soreness scale without an agreed minimally important difference.

### 4.2 Interpretation of effects on subsequent endurance performance

The subsequent endurance-performance findings were mixed, non-poolable, and of very low certainty. Armstrong et al. (2015) reported greater improvement in treadmill time to exhaustion after marathon running in participants allocated to 48 hours of compression socks compared with placebo socks. Brophy-Williams et al. (2017) reported a smaller decrement between repeated 5-kilometre treadmill time trials after compression-sock recovery, and Driller and Halson (2013) reported better maintenance of cycling performance after a 60-minute recovery period with lower-body compression garments. de Glanville and Hamlin (2012) reported improved second 40-kilometre cycling time-trial performance after 24 hours of compression compared with a similar-looking placebo garment; however, the available estimates were based on 90% confidence limits and magnitude-based statistics, and raw condition-level data were unavailable for meta-analysis. These results should therefore be interpreted as favorable signals from individual studies rather than as evidence of a consistent or generalizable recovery-only performance effect.

The performance evidence remains fragmented. The studies differed not only by sport modality, but also by the physiological demands of the initial exercise, the duration of recovery, the performance endpoint used, the comparator condition, and the reporting format. Short-turnaround studies evaluated recovery over 60 minutes, de Glanville and Hamlin evaluated a 24-hour cycling recovery interval, and Armstrong et al. assessed treadmill performance 14 days after marathon running. These time windows likely represent different recovery processes and should not be treated as interchangeable. In addition, Williams et al. (2021) reported improved 8-kilometre cycling time-trial performance with high-pressure compression, but the garment was worn during both the recovery period and the subsequent time trial. That design is informative for multiday exercise with continued garment use, but it does not isolate the recovery effect targeted by the present review. Accordingly, this result should be interpreted as evidence from a mixed recovery-plus-performance-wear design rather than as evidence that post-exercise recovery compression alone improved subsequent cycling performance.

The current findings also help clarify why earlier reviews have reached mixed conclusions. Reviews that combine garments worn during exercise, after exercise, and during subsequent testing may obscure whether observed effects reflect recovery, acute mechanical or perceptual effects during performance, or both (Engel et al., 2016; Weakley et al., 2022; Wang et al., 2025). In contrast, the present review applied a narrower recovery-focused framework. Within that framework, the performance evidence remains limited to favorable and non-favorable signals from individual studies that were too sparse, heterogeneous, and methodologically limited to support a pooled estimate or a confident inference about a recovery-only performance effect.

### 4.3 Influence of garment characteristics, timing, and comparator choice

The included studies varied considerably in garment coverage, pressure, and duration of wear. Interventions ranged from below-knee compression socks and calf stockings to lower-body tights, thigh-high stockings, and full-body compression garments. Wear duration ranged from 60 minutes between exercise bouts to 72 hours after marathon running. Applied pressures were measured directly in some studies but were manufacturer-estimated or based on prior observations in others. This variability is important because compression garments are not a uniform intervention; the physiological and perceptual effects of a below-knee sock worn for 60 minutes may differ from those of full-leg or full-body compression worn overnight or for several days.

Comparator choice was a particularly important source of interpretive uncertainty. Only a minority of included studies used placebo or sham garment comparators that attempted to control for expectancy and sensory feedback, and those garment-type placebo studies contributed performance rather than soreness outcomes to the quantitative syntheses. Within the soreness syntheses, most comparisons used no compression, usual clothing, or loose-fitting clothing, whereas Hill et al. (2014a) used sham ultrasound rather than a sensorially equivalent placebo garment. Therefore, participants in most soreness comparisons were likely aware of whether they had received compression. This is important because soreness was a subjective athlete-reported outcome, and any pooled soreness benefit may partly reflect expectation, sensory feedback, or contextual effects rather than a purely physiological recovery effect. The review findings therefore support cautious interpretation of subjective recovery benefits, particularly where unblinded participants rated soreness after wearing a distinguishable intervention.

The heterogeneity in compression protocols also limits inference about dose-response relationships. Williams et al (Williams et al., 2021). suggested that higher-pressure compression may be more effective than lower-pressure compression for cycling performance in a multiday exercise model, but this finding cannot be generalized directly to recovery-only use because the garment was also worn during the performance test. Across the broader evidence base, there were too few comparable studies to determine whether pressure, body coverage, garment type, or wear duration modifies effects. Future trials should therefore report interface pressure, pressure-gradient characteristics, sizing procedures, adherence, and comfort in sufficient detail to permit intervention-level synthesis.

### 4.4 Risk of bias, certainty of evidence, and confidence in the findings

The interpretation of both co-primary outcomes is constrained by methodological limitations. Most outcome-level risk-of-bias assessments were judged as high risk, and the remaining assessment raised some concerns. The most influential limitation was outcome measurement. For soreness, this reflected the combination of a subjective athlete-reported outcome and limited or poorly credible blinding. For performance, this reflected effort-dependent outcomes assessed under recovery conditions that could often be distinguished by participants. These issues are especially important in compression-garment research because participants can often identify whether they are wearing a compressive garment, and perceived recovery outcomes are vulnerable to expectancy effects. Accordingly, the apparent pooled soreness benefit should be interpreted cautiously because it may partly reflect expectation or contextual effects rather than recovery-specific physiological effects.

The certainty of evidence was very low for subsequent endurance performance and for the soreness syntheses. In GRADE, risk of bias was downgraded as very serious for all outcomes, primarily because 10 of 11 outcome-level assessments were judged at high risk and because the key pooled soreness outcome was subjective and often not credibly blinded. The soreness outcomes were also downgraded for inconsistency and imprecision. Differences in exercise stimulus, comparator credibility, anatomical soreness site, elicitation task, scale range, and recovery protocol were treated primarily as inconsistency or clinical heterogeneity rather than indirectness because the pooled studies still directly assessed eligible self-reported post-exercise soreness in adult endurance athletes within the prespecified recovery windows. By contrast, subsequent endurance performance was downgraded for indirectness because one performance study used a mixed recovery-plus-performance-wear design and the recovery-only effect could not be isolated. Publication bias was not formally assessed because each synthesis included fewer than 10 studies.

The very low certainty does not mean that compression garments are ineffective. Rather, it means that the current evidence is insufficiently robust to estimate the effect with confidence. The available studies generally suggest possible benefits for perceived soreness and, in selected designs, subsequent endurance performance. However, because the evidence base is small, heterogeneous, and often at high risk of bias, these apparent benefits remain uncertain. This distinction is essential for a balanced conclusion and for avoiding overstatement in clinical or athletic recommendations.

### 4.5 Evidence distribution, research gaps, and future research priorities

The evidence map showed concentration in runners and cyclists from Europe and Oceania, with no included evidence from several world regions. Evidence was densest for lower-body garments and below-knee socks or stockings, whereas thigh-high stockings and full-body compression garments were represented by single or sparse evidence units. Soreness outcomes were more frequently reported than cleanly isolatable endurance-performance outcomes. This distribution indicates that the literature has focused more on perceptual recovery than on standardized, recovery-specific performance restoration.

Several research gaps follow directly from this distribution. Adequately powered trials are needed in which compression is applied only during the post-exercise recovery period and removed before the subsequent performance test. This design is necessary to isolate recovery effects from acute effects during exercise. Studies should align outcome assessment with prespecified recovery windows, particularly the approximately-24-hour window that is practically relevant for repeated training and competition. Future trials should use credible sham or placebo garment comparators whenever possible, especially for soreness and perceived recovery outcomes. Crossover studies should report paired data, within-participant correlations, and period or carryover assessments to permit appropriate synthesis. Researchers should define and report minimally important differences for soreness and endurance-performance outcomes so that standardized effects can be interpreted clinically.

Future studies should also broaden participant representation. Several included studies enrolled only male athletes, and sample sizes were generally small. More evidence is needed in female athletes, mixed-sex cohorts with sex-stratified analyses, different endurance sports, and athletes from regions outside Europe and Oceania. Intervention reporting should follow a reproducible template, including garment brand and model, coverage, material, pressure, pressure measurement method, sizing, timing, duration, adherence, thermal comfort, and co-interventions. Such reporting would make it possible to determine whether garment pressure, coverage, or wear duration explains variation in recovery effects.

### 4.6 Practical implications

For athletes and practitioners, the findings do not support a general recommendation for post-exercise compression garments; at most, compression may be trialed as an athlete-specific, low-complexity adjunct when the goal is to manage perceived soreness, provided expectations remain conservative. The pooled soreness estimates favored compression, yet the evidence was very uncertain and confidence intervals crossed the null effect. Therefore, compression garments should not be presented as an evidence-established method for improving soreness recovery in endurance athletes. Their use may be reasonable only as an individualized trial when athletes perceive benefit, tolerate the garment well, and do not substitute compression for recovery strategies with stronger mechanistic or contextual justification, such as adequate sleep, nutrition, hydration, and training-load management.

The findings do not support a strong recommendation that post-exercise compression improves subsequent endurance performance. Some individual studies reported favorable performance outcomes, but these findings were not sufficiently comparable for quantitative synthesis and should not be interpreted as evidence of an established performance benefit. Several designs also had important interpretive limitations, including mixed recovery-plus-performance-wear exposure or incomplete reporting of extractable condition-level data. In applied contexts, compression may be tested individually as part of an athlete-specific recovery routine, particularly during congested training or competition periods, but performance claims should be framed as uncertain. Practitioners should also consider comfort, thermal burden, garment fit, and athlete preference, because discomfort may reduce adherence and may outweigh any perceived recovery benefit for some athletes.

### 4.7 Strengths and limitations of the review

This review has some strengths. It was guided by a prospective protocol that prespecified the population, intervention, comparators, co-primary outcomes, time windows, and synthesis approach. The review separated post-exercise compression from compression worn during exercise or during subsequent performance, which addresses an important source of heterogeneity in prior evidence syntheses. The extraction framework also distinguished study-level eligibility from outcome-level synthesis eligibility, allowing studies with relevant but non-poolable evidence to be included narratively without being combined inappropriately.

However, the review also has limitations. The final synthesis depends on the completeness and accuracy of published reports, and several studies did not report all statistics required for optimal paired or time-windowed meta-analysis. The search was conducted in PubMed, Scopus, and Web of Science, supplemented by reference-list and forward-citation checking, but it did not include every possible database, trial registry, dissertation database, conference-proceedings source, or grey-literature source. SPORTDiscus, ClinicalTrials.gov, WHO ICTRP, dissertation databases, and grey-literature sources were not searched in the original strategy. Therefore, unpublished, or registered but unpublished may have been missed. This is important because publication bias could not be formally assessed with fewer than 10 studies per synthesis, and recent running-focused syntheses have reported overall null or non-supportive findings for performance, physiological, or perceptual outcomes.

## 5 Conclusions

This systematic review with meta-analysis found very low-certainty evidence on post-exercise compression garments for delayed-onset or perceived muscle soreness and subsequent endurance performance in endurance athletes. Soreness estimates at approximately 24 hours and immediate to 2 hours were directionally favorable to compression, but they were statistically uncertain, imprecise, and vulnerable to expectation effects because most soreness comparisons used unblinded or poorly blinded subjective athlete-reported outcomes. Several individual studies reported favorable subsequent endurance-performance outcomes, but the evidence was too heterogeneous by recovery window, sport modality, intervention protocol, reporting format, and isolation of the recovery effect to support a pooled estimate or a confident recovery-only performance inference. Current evidence is therefore insufficient to make strong effectiveness claims. Future well-designed, adequately blinded, and transparently reported trials are needed to determine whether post-exercise compression garments meaningfully improve soreness recovery or subsequent endurance performance, and under which garment and recovery conditions.

## Data Availability

The original contributions presented in the study are included in the article/**Supplementary Material**. Further inquiries can be directed to the corresponding author.
