# Supplementary material for: Effects of post-exercise compression garments on subsequent endurance performance and delayed-onset muscle soreness in endurance athletes: a systematic review and meta-analysis
Source: Front Physiol. 2026 Jul 31;17:1908148. doi: 10.3389/fphys.2026.1908148 (PMC13472931; doi:10.3389/fphys.2026.1908148)
Supplement: Supplementary file 1 [file Table1.docx]

Supplementary Table S1. Extracted soreness values and scale context.

| Study | Window | Comparator | Outcome/task and scale | Compression value | Comparator value | Baseline/pre-recovery |
| --- | --- | --- | --- | --- | --- | --- |
| Bieuzen et al. 2014 | 1 h; 24 h | No compression; unblinded subjective outcome | Half-squat soreness; 10-cm VAS | 1 h: 5.0 ± 1.9; 24 h: 4.2 ± 1.9 | 1 h: 4.8 ± 2.6; 24 h: 4.1 ± 1.9 | Arm-level follow-up values; recovery compression was slightly worse than no compression at both time points. |
| Brophy-Williams et al. 2017 | Immediate-to-2 h | No compression; unblinded subjective outcome; all had prior compression experience | Half-squat soreness; 10-point VAS | Pre: 4.8 ± 2.05; post: 2.8 ± 1.10 | Pre: 3.8 ± 1.89; post: 4.0 ± 2.05 | Pre-recovery soreness was higher in compression; post-recovery values selected. |
| Driller and Halson 2013 | Immediate-to-2 h | Loose-fitting shorts; unblinded subjective outcome | Perceived soreness; 1-10 scale | Pre: 7.4 ± 1.9; post: 1.9 ± 1.5 | Pre: 6.5 ± 2.4; post: 3.2 ± 2.5 | Pre-recovery soreness was slightly higher in compression; post-recovery values selected for synthesis. |
| Hill et al. 2014 | Approximately 24 h | Sham ultrasound; non-garment sham; subjective outcome | Squat soreness; 200-mm VAS | Immediate: 31.0 mm; 24 h: 13.9 ± 12.9 mm | Immediate: 26.8 mm; 24 h: 36.4 ± 11.9 mm | Parallel groups differed in age; SDs for 24 h were digitized from Figure 1. |
| Li et al. 2026 | Approximately 24 h | No compression/usual clothing; unblinded subjective outcome | DOMS VAS; 0-100 mm | Day 1 Post: 58.17 ± 26.01; Day 2 Pre: 38.83 ± 21.00 | Day 1 Post: 51.00 ± 20.60; Day 2 Pre: 46.25 ± 25.06 | Direction unfavorable at Day 1 Post but favorable at Day 2 Pre; Day 2 Pre selected by time-window hierarchy. |
| Martínez-Navarro et al. 2021 | Approximately 24 h | No compression; unblinded subjective outcome | Posterior-leg DOMS; 10-cm VAS reported as % change from post-race value | 11.0 ± 46.2% | 112.3 ± 170.4% | 112.3% extracted from abstract and supported by Figure 1. |

Supplementary Table S2. Effect-estimate coding and model inputs.

| Study | Window | Design basis | Direction | Hedges g | SE | Variance | Assumed r |
| --- | --- | --- | --- | --- | --- | --- | --- |
| Bieuzen et al. 2014 | 1 h | Crossover; arm-level follow-up values | Positive = lower soreness with compression; value coded negative because compression > control | -0.08 | 0.279 | 0.0778 | 0.50 |
| Bieuzen et al. 2014 | 24 h | Crossover; arm-level follow-up values | Positive = lower soreness with compression; value coded negative because compression > control | -0.05 | 0.279 | 0.0776 | 0.50 |
| Brophy-Williams et al. 2017 | Immediate-to-2 h | Crossover; arm-level post-recovery values | Positive = lower soreness with compression | 0.68 | 0.302 | 0.0913 | 0.50 |
| Driller and Halson 2013 | Immediate-to-2 h | Crossover; arm-level post-recovery values | Positive = lower soreness with compression | 0.58 | 0.317 | 0.1002 | 0.50 |
| Hill et al. 2014 | 24 h | Parallel-group follow-up values | Positive = lower soreness with compression | 1.75 | 0.485 | 0.2351 | Not applicable |
| Li et al. 2026 | 24 h | Crossover; Day 2 pre-exercise values | Positive = lower soreness with compression | 0.30 | 0.275 | 0.0758 | 0.50 |
| Martínez-Navarro et al. 2021 | 24 h | Parallel-group percentage-change values | Positive = lower soreness with compression | 0.79 | 0.368 | 0.1354 | Not applicable |
